# Design, Synthesis, and Biological Evaluation of Novel Thienopyrimidine Derivatives as PI3Kα Inhibitors

**DOI:** 10.3390/molecules24193422

**Published:** 2019-09-20

**Authors:** Lide Yu, Qinqin Wang, Caolin Wang, Binliang Zhang, Zunhua Yang, Yuanying Fang, Wufu Zhu, Pengwu Zheng

**Affiliations:** 1 Jiangxi Provincial Key Laboratory of Drug Design and Evaluation, School of Pharmacy, Jiangxi Science & Technology Normal University, 605 Fenglin Road, Nanchang 330013, China; 20020680@jxutcm.edu.cn (L.Y.); m15180134379_2@163.com (Q.W.); wangcllw@163.com (C.W.); zbl1045762244@163.com (B.Z.); 2 College of Pharmacy, Jiangxi University of Traditional Chinese Medicine, Nanchang 330004, China; mtdzcool@163.com (Z.Y.); fangyuanying@163.com (Y.F.)

**Keywords:** Thienopyrimidine, Pyrazole, PI3Kα inhibitor

## Abstract

Three series of novel thienopyrimidine derivatives **9a**–**l**, **15a**–**l**, and **18a**–**h** were designed and synthesized, and their IC_50_ values against four cancer cell lines HepG-2, A549, PC-3, and MCF-7 were evaluated. Most compounds show moderate cytotoxicity against the tested cancer cell lines. The most promising compound **9a** showed moderate activity with IC_50_ values of 12.32 ± 0.96, 11.30 ± 1.19, 14.69 ± 1.32, and 9.80 ± 0.93 µM, respectively. The inhibitory activities of compounds **9a** and **15a** against PI3Kα and mTOR kinase were further evaluated. Compound **9a** exhibited PI3Kα kinase inhibitory activity with IC_50_ of 9.47 ± 0.63 µM. In addition, docking studies of compounds **9a** and **15a** were also investigated.

## 1. Introduction

**Figure 1:**
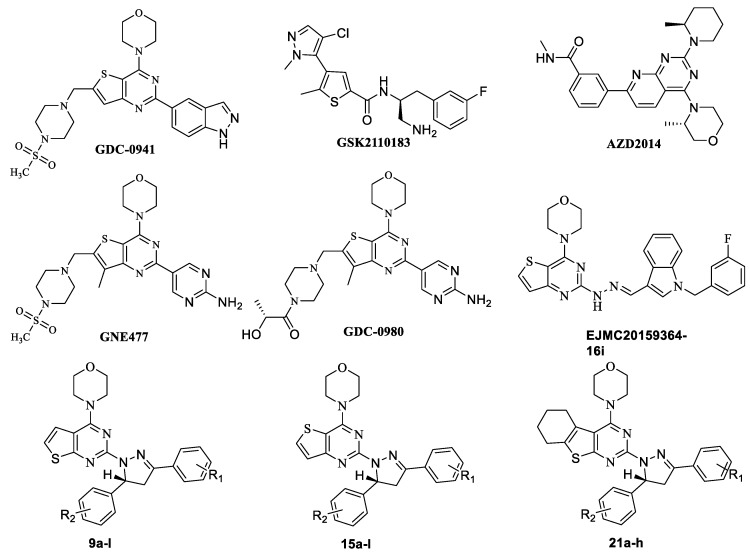
Structures of representative clinical PI3K/mTOR dual inhibitors and target compounds.

The PI3K-Akt-mTOR signaling pathway plays an important role in tumorigenesis and development [1,2]. The activation of related proteins in this pathway is closely related to the occurrence and development of tumors. In recent years, the development of small molecule drugs to effectively inhibit the overexpression of this pathway has become a research hotspot in cancer therapy [3]. Moreover, the study of the complex co-crystals structure of protein and small molecular Ligand also promoted the development of new drugs [4,5,6]. Many small molecule inhibitors targeting PI3K-Akt–mTOR signaling pathway have entered clinical studies, and some of them were even approved by FDA, such as PI3K inhibitor GDC-0941 [7]; AKT inhibitor GSK2110183 [8]; mTOR inhibitor AZD2014 (Figure 1) [9], Rapamycin, Deforolimus. PI3Kα is the most important isoform in cell proliferation in response to growth factor-tyrosine kinase pathway activation. There are currently more than ten PI3Kα inhibitors undergoing clinical trials. Many research groups are attempting to develop some more PI3Kα inhibitors. 

The thienopyrimidine core is widely used in small molecule inhibitors of the PI3K-Akt-mTOR signaling pathway. Among them, thienopyrimidine derivative GDC-0941 developed by Genentech, is the first PI3K inhibitor entering the clinical stage. Right after, several analogues of GDC-0941 were obtained. Two of them, GNE-477 and GDC-0980 (Figure 1), exhibited excellent activities and were regarded as potent PI3K/mTOR inhibitors [10,11]. Research showed that thienopyrimidines core were very important to the activity of these compounds, and it was considered to be an active pharmacophore.

**Figure 2:**
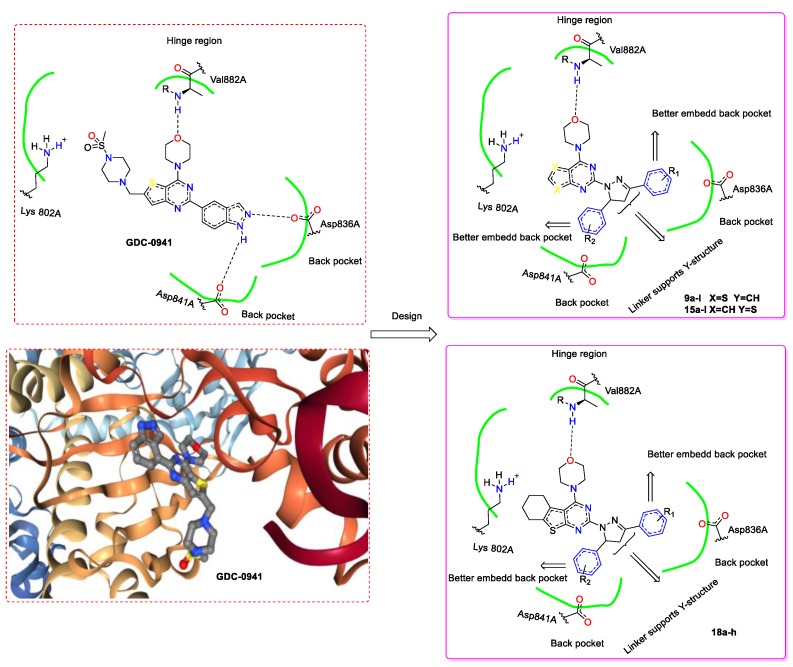
The design concept based on the co-crystal structure of GDC-0941 with protein.

In our previous research, several series of thienopyrimidine-containing compounds were designed and synthesized [12,13,14], one representative compound EJMC20159364-16i (Figure 1) exhibited the best in vitro cytotoxic activity and kinase inhibitory activity (PI3K, mTOR). SARs of this series of compounds were summarized. The results showed that the introduction of the flexible hydrazinyl linker at the pyrimidine 2 position helps to increase the cytotoxic activity of the target compounds. Continuous to this work, the hydrazinyl linker was kept unchanged in the new designed compounds, and we constructed a pyrazole ring on the hydrazinyl as a new flexible linker to connect with the aryl group, hoping to increase the compounds’ inhibitory activity by improving the interaction of the compounds with the receptor (Figure 2). As a result, two series of thieno[2,3-*d*]pyrimidines and thieno[3,2-*d*]pyrimidines containing pyrazole unit (**9a**–**l**, **15a**–**l**) were designed and synthesized. Inspired by GDC-0084, in order to investigate the influence of the thienopyrimidine core to the activity, a flexible cyclohexane was introduced to the target compounds, resulting in a series of tricyclic thienopyrimidine compounds **21a**–**h**. It is expected to increase the cytotoxic activity and increase the interaction of the compounds with the enzyme to enhance the activity and selectivity. Finally, three series of thienopyrimidine derivatives containing pyrazole structure (**9a**–**l**, **15a**–**l, 21a**–**h**) were designed, synthesized and evaluated for their cytotoxic activity.

## 2. Results and Discussion

### 2.1. Chemistry

**Scheme 1:**
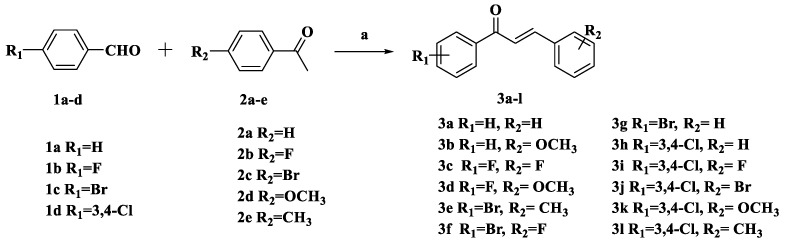
Synthetic routes of chalcones **3a**–**l**. Reagents and conditions: **a** 10% NaOH, EtOH, r.t., 24 h.

**Scheme 2:**
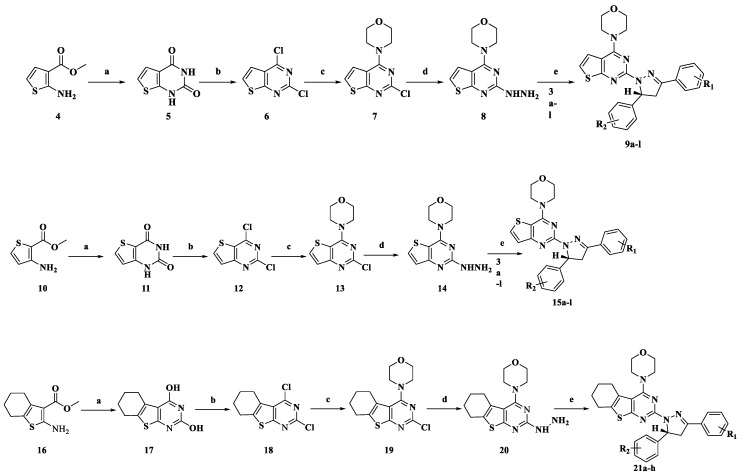
Synthetic routes of target compounds **9a**–**l**, **15a**–**l**, and **21a**–**h**. Reagents and conditions: **a** 5 eq urea, 180 °C, 2h; **b** POCl_3_, DMF (cat.), reflux, 8 h; **c** 2.1 eq morpholine, MeOH, 0 °C, 30 min, r.t., 2h; **d** 80% hydrazine monohydrate, reflux, 8h; **e** Glacial acetic acid, H_2_SO_4_ (78%), 100 °C.

The synthetic routes for thienopyrimidine derivatives **9a**–**l**, **15a**–**l,** and **21a**–**h** are outlined in Scheme 1 and Scheme 2. Condensation of the 4-substituted benzaldehydes (**1a**–**d**) with the 4-substituted acetophenones (**2a**–**e**) produced the corresponding chalcones **3a**–**l** (Scheme 1). Treatment of **5** with POCl _3_ afforded 2,4-dichlorothieno[2,3-*d*]pyrimidine **6**, which was then treated with morpholine to give 4-(2-chlorothieno[2,3-*d*]pyrimidin-4-yl)morpholine **7**. Treatment of **7** with hydrazine gave the key intermediate **8**. Intermediate **8** condensed with the corresponding chalcones **3a**–**l** to afford the target compounds **9a**–**l**. The synthesis of compounds **15a**–**l** and **21a**–**h** was similar to that of compounds **9a**–**l** (Scheme 2). Generated by condensation reaction between **8** chalcone **3a**–**l,** there appears a chiral carbon atom at C-5 position of pyrazole of all of the compounds. The similar reaction was reported in previous research and the structures of target compounds can be easily identified. The structures of target compounds were confirmed by ^1^H-NMR, ^13^C-NMR, and TOF MS (ESI+), which were in agreement with the structures depicted.

### 2.2. Biological Discussion

All target compounds were evaluated for their cytotoxic activities against A549 (human lung cancer), PC-3 (human prostate cancer), MCF-7 (human breast cancer), HepG2 (human hepatoma) cell lines. In addition, the activity against PI3Kα/mTOR kinase of the most promising compounds **9a** and **15a** was further evaluated. The results expressed as IC_50_ were summarized in Table 1, Table 2 and Table 3.

As illustrated in Table 1 and Table 2, most of the target compounds exhibited moderate cytotoxic activities. The compounds whose skeleton are thieno[2,3-*d*]pyrimidine (**9a**–**1**) has slightly stronger inhibitory activity against the tested cancer cell lines than the compounds whose skeleton are thieno[3,2-*d*]pyrimidine (**15a**–**l** and **21a**–**h**). The most promising compound **9a** showed moderate to well inhibitory activity with IC_50_ values of 12.32 ± 0.96, 11.30 ± 1.19, 14.69 ± 1.32, and 9.80 ± 0.93 µM, respectively.

It is worth noting that when R_1_ of the aryl group is an electron-withdrawing group (EWG) and R_2_ is a H atom, the inhibitory activity of the compounds (**9g**–**h**, **15g**–**h,** and **21a**–**h**) against cancer cells is decreased, especially for MCF-7 and A549 cells. The effect is greater than that of HepG2 and PC-3 cancer cell lines. Among them, the inhibitory activity of the compound **15h** against MCF-7 cells was IC_50_ > 50 μM, which means that the inhibitory activity was almost lost. When the C-3 and C-4 positions of the aryl group were both replaced by chlorine, the activity of the compounds (**9h**–**l**, **15h**–**l**) is slightly lower than that of the compounds (**9b**–**g**, **15b**–**g**) substituted by F or Br atoms.

When R_1_ of the aryl group was hydrogen atom and R_2_ was an electron-donating group, the inhibitory activities of the compounds **9b** and **15b** against the tested cancer cells decreased. The compound **15b** which contained the core skeleton thieno[3,2-*d*]pyrimidine was more obvious in this respect, and its IC _50_ values for A549 and MCF-7 were both higher than 50 μM. When R _1_ of the aryl group was an electron withdrawing group, the inhibitory activity against cancer cells of the compounds **9c**–**l** and **15c**–**l** tended to decrease irrespective whether the substituent of R_2_ was an electron withdrawing group or an electron donating group. Comparing the difference in activity between **9e**–**f** and **15e**–**f**, it is easily to find that the effect of the electron withdrawing group on the activity is slightly less than that of the electron-donating group. As shown in Table 3, compounds **21a**–**h** exhibited poor cytotoxic activity against the tested cell lines. This may attributed to the introduction of the tricyclic thieno-pyrimidine structure, resulting in an increase in steric hindrance which reduces the solubility of the compound at physiological pH and the relative molecular mass of the compound exceeds 500.

Finally, the inhibitory activity against PI3Kα kinase and mTOR kinase of selected compounds **9a** and **15a** were further examined. As shown in Table 3, enzymatic activity results of compounds **9a** and **15a** exhibited a moderate to excellent inhibitory activity against PI3Kα kinase. The inhibitory activity of the compounds against PI3Kα kinase is better than that of mTOR kinase.

### 2.3. Molecular Docking Study of Compounds **9a** and **15a**.

**Figure 3:**
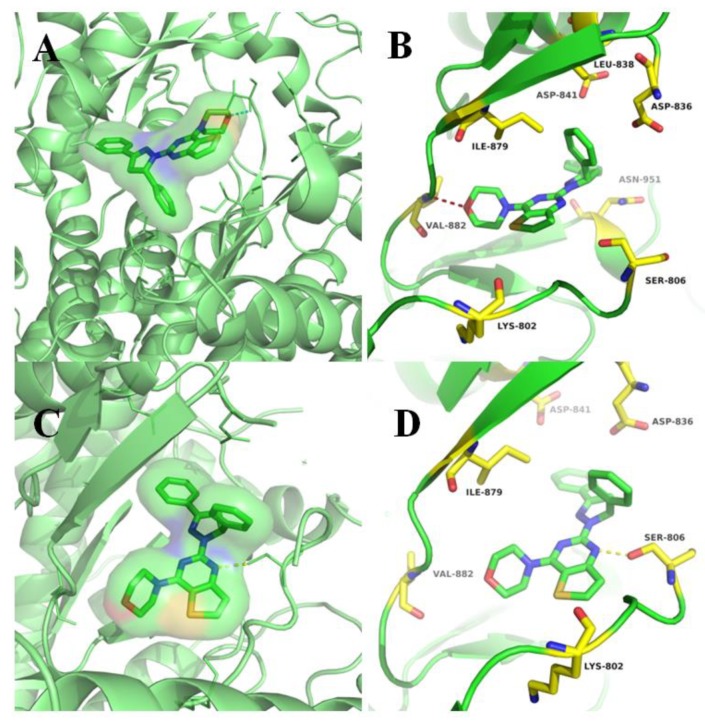
Docking modes of **9a** and **15a**. (**A**) Overview of the binding site of compound **9a** with PI3Kα (3TL5) kinase. (**B**) Binding model of compound **9a** with PI3Kα (3TL5) kinase. (**C**) Overview of the binding site of compound **15a** with PI3Kα (3TL5) kinase. (**D**) Binding model of compound **15a** with PI3Kα (3TL5) kinase.

To further explore the binding modes of target compounds (**9a** and **15a**) with the active site of PI3Kα, molecular docking simulation studies were carried out by the AutoDock 4.2 software. Based on in vitro inhibition results, we selected compounds **9a** and **15a** as ligand examples, and the structures of PI3Kα (PDB code: 3TL5) were selected as the docking models. The best-scoring ligand–protein complex (Figure 3) was used for binding site analysis.

The binding models of compounds **9a** and **15a** to the 3TL5 protein are approximately the same as our previously assumed binding model (Figure 3A,C). The detailed binding models of compounds **9a** and **15a** with the active site of PI3Kα kinase are shown in Figure 3. In the docking model of compound **9a** with PI3Kα (Figure 3B), we can easily see that the oxygen atom on the morphine ring formed a hydrogen bond with the hinge region residue VAL882. After careful observation of the binding model of compound **15a** with 3TL5 protein kinase (Figure 3D), it is not difficult to find that the N atom on the pyrimidine ring of thieno[3,2-*d*]pyrimidine formed a hydrogen bond interaction with residue SER806. Compared with the original ligand GDC-0980, the hydrogen bonding interaction between compounds **9a** and **15a** with protein is reduced, which may be one of the reasons why this series of compounds failed to achieve excellent cytotoxic activity.

## 3. Experimental Section

### 3.1. General Information

Unless otherwise required, all reagents used in the experiment were purchased as commercial analytical grade and used without further purification. Frequently used solvents (Ethanol, petroleum ether, ethyl acetate, dichloromethane, etc.) were absolutely anhydrous. All actions were monitored through GF_254_ thin-layer chromatography plate (Qingdao Haiyang Chemical Co., Ltd., Qingdao, China) and spots were visualized with iodine or light (in 254 nm or 365 nm). The structure of the target compound was confirmed by ^1^H-NMR and ^13^C-NMR spectra at room temperature on Bruker 400 MHz spectrometer (Bruker Bioscience, Billerica, MA, USA) with tetramethylsilane (TMS) as an internal standard. Mass spectrometry (MS) was performed on Waters High Resolution Quadrupole Time of Flight Tandem Mass Spectrometry (QTOF) (Waters Corporation, Milford, MA, USA). The purity of the compound was determined by Agilent 1260 liquid chromatograph (Agilent Technologies Inc., Palo Alto, CA, USA) fitted with an Inertex-C18 column. All target compounds had the purity of ≥95%.

### 3.2. Chemistry

#### 3.2.1. General Procedure for the Preparation of Compounds **3a**–**l**

Compounds **3a**–**l** were synthesized according to the reported procedures by our research group [15].

#### 3.2.2. General Procedure for the Preparation of Compounds **8**, **14** and **20**

Compound **5** was obtained by a cyclization reaction starting from commercially available methyl 2-aminothiophene-3-carboxylate (**4**) and urea. Subsequently, compound **5** is subjected to a chlorination reaction with POCl_3_ to give compound **6**. Compound **6** was substituted by morphine and hydrazine hydrate respectively to obtain key intermediate **8**. Similarly, we used commercially available methyl 2-aminothiophene-3-carboxylate (**10**) or commercial available methyl 2-amino-4,5,6,7-tetrahydrobenzo[*b*]thiophene-3-carboxylate (**16**) as the starting material to obtain intermediates **14** or **20** through a similar reaction condition. Detailed synthesis of key intermediates **8**, **14**, and **20**, and their precursors **7**, **11**, **12**, and **13**. can be found in the article reported by our research group [13].

#### 3.2.3. General Procedure for the Preparation of Target Compounds **9a**–**l**, **15a**–**l**, and **21a**–**h**.

A mixture of different substituted chalcone **3a**–**l** (1.5 mmol) and 4-(2-mercaptothieno[2,3-*d*]pyrimidin-4-yl) morpholine (**8**) or 4-(2-mercaptothieno[3,2-*d*]pyrimidin -4-yl)morpholine (**14**) or 4-(2-hydrazinyl-5,6,7,8-tetrahydrobenzo[4,5]thieno[2,3-*d*]pyrimidin-4-yl)morpholine (**20**) were dissolved (1.5 mmol, **8**/**14**/**20**) in 25 mL of glacial acetic acid. Subsequently, the reaction solution was stirred at 100–110 °C for 1.5 h under the action of 2–3 drops concentrated H_2_SO_4_ (78%) as a catalyst and monitored by thin-layer chromatography (TLC). The reaction mixture was concentrated under reduced pressure. After the completion of concentrated, the mixture was filtered and the precipitate was washed with ethanol. If the purify of the compound is not high enough, recrystallization by 95% EtOH or column chromatography (EtOAc:PE = 1:3) are needed. The obtained solids were then dried to give the target compounds **9a**–**l**, **15a**–**l**, and **18a**–**h** with the yield ranging from 30%–60%.

*4-(2-(3-(3,4-dichlorophenyl)-5-(4-methoxyphenyl)-4,5-dihydro-1H-pyrazol-1-yl)thieno[2,3-d]pyrimidin-4-yl)morpholine* (**9a**). Light yellow solid, mp 202–205 °C; ^1^H NMR (400 MHz, DMSO-*d_6_*) δ (ppm): 8.42 (d, *J* = 5.3 Hz, 1H), 7.95 (d, *J* = 8.7 Hz, 2H), 7.73 (s, 1H), 7.62 (m, *J* = 16.2, 7.0 Hz, 2H), 7.33 (d, *J* = 8.1 Hz, 1H), 7.12 (d, *J* = 8.4 Hz, 2H), 5.85–5.75 (m, 1H), 4.08 (m, *J* = 17.7, 11.6 Hz, 1H), 3.85 (s, 3H), 3.84–3.75 (m, 3H), 3.67 (s, 3H), 3.60–3.54 (m, 1H), 3.50–3.46 (m, 2H). HRMS (ESI): *m*/*z* calcd for (C_27_H_25_N_3_OS + H)**^+^**: 440.1797; found: 440.1834.

*4-(2-(5-(4-bromophenyl)-3-(3,4-dichlorophenyl)-4,5-dihydro-1H-pyrazol-1-yl)thieno[2,3-d]pyrimidin-4-yl)morpholine* (**9b**). Light yellow solid, m.p. 280–282 °C; ^1^H NMR (400 MHz, DMSO-*d_6_*) δ (ppm): 8.18 (d, *J* = 5.5 Hz, 1H), 7.90 (dd, *J* = 8.4, 5.5 Hz, 2H), 7.63 (dd, *J* = 8.4, 5.0 Hz, 2H), 7.37 (dd, *J* = 13.9, 7.0 Hz, 3H), 7.30–7.26 (m, 1H), 5.79 (dd, *J* = 12.1, 6.1 Hz, 1H), 3.95 (dd, *J* = 17.8, 12.2 Hz, 1H), 3.82–3.65 (m, 6H), 3.59 (d, *J* = 7.7 Hz, 3H). HRMS (ESI): *m*/*z* calcd for (C_28_H_27_N_3_O_2_S + H)**^+^**: 470.1902; found: 470.1946.

*4-(2-(3,5-bis(4-fluorophenyl)-4,5-dihydro-1H-pyrazol-1-yl)thieno[2,3-d]pyrimidin-4-yl)Morpholine* (**9c**).Yellow solid, m.p. 225–227 °C; ^1^H NMR (400 MHz, DMSO-*d_6_*) δ (ppm): 8.47 (d, *J* = 5.6 Hz, 1H), 8.13 (s, 2H), 7.63 (s, 1H), 7.50–7.42 (m, 4H), 7.25 (t, *J* = 8.5 Hz, 2H), 5.88 (s, 1H), 4.13 (d, *J* = 12.1 Hz, 1H), 3.87 (s, 4H), 3.70 (s, 3H), 3.49 (s, 1H), 3.22 (s, 1H). HRMS (ESI): *m*/*z* calcd for (C_27_H_23_F_2_N_3_OS + H)**^+^**: 476.1608; found: 476.1598.

*4-(2-(3-(3,4-dichlorophenyl)-5-(4-fluorophenyl)-4,5-dihydro-1H-pyrazol-1-yl)thieno[2,3-d]pyrimidin-4-yl)morpholine* (**9d**).Yellow solid, m.p. 286–289 °C; ^1^H NMR (400 MHz, DMSO-*d_6_*) δ (ppm): 7.73 (d, *J* = 7.5 Hz, 2H), 7.54 (d, *J* = 7.9 Hz, 2H), 7.31 (d, *J* = 6.6 Hz, 3H), 7.25 (d, *J* = 7.8 Hz, 2H), 5.74 (d, *J* = 6.6 Hz, 1H), 3.95–3.84 (m, 1H), 3.76–3.63 (m, 6H), 3.59 (s, 2H), 3.49 (d, *J* = 5.9 Hz, 1H). HRMS (ESI): *m*/*z* calcd for (C_28_H_26_FN_3_O_2_S + H)**^+^**: 488.1808; found: 488.1853.

*4-(2-(3-(3,4-Dichlorophenyl)-5-phenyl-4,5-dihydro-1H-pyrazol-1-yl)thieno[2,3-d]pyrimidine-4-yl)morpholine* (**9e**). Yellow solid, m.p. 228.3~229.1 °C; ^1^H NMR (400 MHz, DMSO-*d_6_*) δ (ppm): 8.07 (d, *J* = 5.4 Hz, 1H), 7.77 (d, *J* = 7.1 Hz, 2H), 7.55 (d, *J* = 8.4 Hz, 2H), 7.44 (d, *J* = 7.6 Hz, 3H), 7.25 (d, *J* = 5.5 Hz, 2H), 5.72 (dd, *J* = 12.1, 5.7 Hz, 1H), 3.87 (dd, *J* = 18.0, 12.3 Hz, 1H), 3.71 (s, 2H), 3.66–3.58 (m, 4H), 3.54 (s, 2H), 3.18 (dd, *J* = 18.1, 5.8 Hz, 1H). HRMS (ESI): *m*/*z* calcd for (C_28_H_26_BrN_3_OS + H)**^+^**: 534.1041; found: 534.0991.

*4-(2-(5-(4-methoxyphenyl)-3-phenyl-4,5-dihydro-1H-pyrazol-1-yl)thieno[2,3-d]pyrimidine-4-yl)morpholine* (**9f**). Orange solid, m.p. 237.6~239.1 °C; ^1^H NMR (400 MHz, DMSO-*d_6_*) δ (ppm): 8.17 (d, *J* = 4.9 Hz, 1H), 7.79 (d, *J* = 8.1 Hz, 2H), 7.35 (d, *J* = 4.7 Hz, 1H), 7.29 (d, *J* = 7.3 Hz, 2H), 7.24 (d, *J* = 7.7 Hz, 3H), 7.03 (d, *J* = 8.3 Hz, 2H), 5.69 (d, *J* = 6.4 Hz, 1H), 3.98–3.87 (m, 2H), 3.80 (s, 3H), 3.68 (s, 4H), 3.57 (s, 4H). HRMS (ESI): *m*/*z* calcd for (C_27_H_23_BrFN_3_OS + H)**^+^**: 538.0790; found: 538.0882.

*4-(2-(3-(3,4-dichlorophenyl)-5-(p-tolyl)-4,5-dihydro-1H-pyrazol-1-yl)thieno[2,3-d]pyrimidin-4-yl)morpholine* (**9g**). Yellow solid, m.p. 207.4~208.2 °C; ^1^H NMR (400 MHz, DMSO-*d_6_*) δ (ppm): 8.43 (d, *J* = 5.4 Hz, 1H), 7.90 (d, *J* = 7.6 Hz, 2H), 7.74 (s, 1H), 7.62 (dd, *J* = 15.4, 6.7 Hz, 2H), 7.41–7.29 (m, 3H), 5.80 (dd, *J* = 11.6, 6.2 Hz, 1H), 4.09 (dd, *J* = 18.2, 11.5 Hz, 1H), 3.82 (s, 4H), 3.67 (s, 3H), 3.48 (s, 2H), 2.40 (s, 3H). HRMS (ESI): *m/z* calcd for (C_27_H_24_BrN_3_OS + H)**^+^**: 520.0884; found: 520.0865.

*4-(2-(3-(4-bromophenyl)-5-(p-tolyl)-4,5-dihydro-1H-pyrazol-1-yl)thieno[2,3-d]pyrimidine-4-yl)morpholine* (**9h**). Light yellow solid, m.p. 222.8~223.9 °C; ^1^H NMR (400 MHz, DMSO-*d_6_*) δ (ppm): 8.11 (d, *J* = 5.0 Hz, 1H), 7.73 (d, *J* = 7.5 Hz, 2H), 7.54 (d, *J* = 7.9 Hz, 2H), 7.31 (d, *J* = 6.6 Hz, 3H), 7.25 (d, *J* = 7.8 Hz, 2H), 5.74 (d, *J* = 6.6 Hz, 1H), 3.95–3.84 (m, 1H), 3.76–3.63 (m, 6H), 3.59 (s, 2H), 3.13 (d, *J* = 13.7 Hz, 1H), 2.40 (s, 3H). ^13^C NMR (101 MHz, DMSO-*d_6_*) δ 163.89, 157.95, 156.29, 150.82, 145.81, 134.03, 132.46, 131.45, 131.41, 129.81, 129.71(2,C), 129.17, 128.43, 126.68(2,C), 126.29, 124.43, 106.60, 66.29(2,C), 61.90, 46.26(2,C).HRMS (ESI): *m*/*z* calcd for (C_27_H_23_Cl_2_N_3_OS + H)**^+^**: 508.1017; found: 508.1059.

4-(2-(3,5-diphenyl-4,5-dihydro-1H-pyrazol-1-yl)thieno[2,3-d]pyrimidin-4-yl)morpholine (**9i**). Light yellow solid, m.p. 231.7~233.2 °C; ^1^H NMR (400 MHz, DMSO-*d_6_*) δ (ppm): 8.04 (d, *J* = 5.5 Hz, 1H), 7.77 (d, *J* = 7.6 Hz, 2H), 7.48–7.37 (m, 3H), 7.30–7.17 (m, 6H), 5.71 (dd, *J* = 12.0, 5.3 Hz, 1H), 3.87 (dd, *J* = 17.4, 12.2 Hz, 1H), 3.63 (dt, *J* = 19.1, 10.7 Hz, 6H), 3.49 (d, *J* = 13.7 Hz, 2H), 3.45–3.40 (m, 1H). HRMS (ESI): *m*/*z* calcd for (C_27_H_22_Cl_2_FN_3_OS + H)**^+^**: 528.0898; found: 528.0891.

*4-(2-(3-(4-fluorophenyl)-5-(4-methoxyphenyl)-4,5-dihydro-1H-pyrazol-1-yl)thieno[2,3-d]pyrimidin-4-yl)morpholine* (**9j**). Light yellow solid, m.p. 215.8~216.8 °C; ^1^H NMR (400 MHz, DMSO-*d_6_*) δ (ppm): 7.91–7.86 (m, 2H), 7.50 (t, *J* = 5.0 Hz, 1H), 7.39–7.24 (m, 4H), 7.16 (t, *J* = 8.8 Hz, 1H), 7.06 (d, *J* = 8.6 Hz, 2H), 5.72 (dd, *J* = 11.0, 5.3 Hz, 1H), 4.07–3.96 (m, 1H), 3.82 (s, 3H), 3.74 (s, 4H), 3.65–3.56 (m, 3H), 3.48 (s, 2H). HRMS (ESI): *m*/*z* calcd for (C_27_H_22_BrCl_2_N_3_OS + H)**^+^**: 588.0100; found: 588.0110.

*4-(2-(3-(4-bromophenyl)-5-(4-fluorophenyl)-4,5-dihydro-1H-pyrazol-1-yl)thieno[2,3-d]Pyrimidin-4-yl)morpholine* (**9k**). Yellow solid, m.p. 221.2~222.0 °C; ^1^H -NMR (400 MHz, DMSO-*d_6_*) δ (ppm): 8.14 (d, *J* = 5.6 Hz, 1H), 7.93–7.85 (m, 2H), 7.55 (d, *J* = 8.2 Hz, 2H), 7.39–7.30 (m, 3H), 7.27 (d, *J* = 8.3 Hz, 2H), 5.77 (dd, *J* = 11.9, 5.4 Hz, 1H), 3.93 (dd, *J* = 17.7, 12.3 Hz, 1H), 3.80–3.65 (m, 6H), 3.59 (d, *J* = 10.2 Hz, 2H), 3.19 (dd, *J* = 17.9, 5.8 Hz, 1H). HRMS (ESI): *m*/*z* calcd for (C_28_H_25_Cl_2_N_3_O_2_S + H)**^+^**: 538.1123; found: 538.1199.

*4-(2-(3-(4-bromophenyl)-5-phenyl-4,5-dihydro-1H-pyrazol-1-yl)thieno[2,3-d]pyrimidine-4-Morpholine* (**9l**). Light yellow solid, m.p. 221.2~222.0 °C; ^1^H NMR (400 MHz, DMSO-*d_6_*) δ (ppm): 8.05 (d, *J* = 5.5 Hz, 1H), 7.76 (d, *J* = 7.3 Hz, 2H), 7.50 7.39 (m, 5H), 7.21 (dd, *J* = 17.0, 6.8 Hz, 3H), 5.70 (d, *J* = 6.7 Hz, 1H), 3.86 (d, *J* = 5.2 Hz, 1H), 3.69 (t, *J* = 10.6 Hz, 2H), 3.64–3.57 (m, 4H), 3.53 (t, *J* = 9.8 Hz, 2H), 3.12 (d, *J* = 5.5 Hz, 1H). HRMS (ESI): *m*/*z* calcd for (C_28_H_25_Cl_2_N_3_OS + H)**^+^**: 522.1174; found: 522.1203.

*4-(2-(3-(3,4-dichlorophenyl)-5-(4-methoxyphenyl)-4,5-dihydro-1H-pyrazol-1-yl)thieno[3,2-d]pyrimidin-4-yl)morpholine* (**15a**). Light yellow solid, m.p. 131.2~132.5 °C; ^1^H NMR (400 MHz, DMSO-*d_6_*) δ (ppm): 7.73 (d, *J* = 8.4 Hz, 2H), 7.57 (d, *J* = 8.1 Hz, 2H), 7.40 (d, *J* = 6.2 Hz, 1H), 7.19 (d, *J* = 6.0 Hz, 2H), 7.02 (d, *J* = 8.5 Hz, 2H), 5.69 (dd, *J* = 12.0, 5.5 Hz, 1H), 3.92–3.84 (m, 1H), 3.81 (s, 3H), 3.62 (dd, *J* = 22.9, 8.6 Hz, 6H), 3.51 (d, *J* = 8.6 Hz, 2H), 3.17 (dd, *J* = 17.7, 5.6 Hz, 1H). HRMS (ESI): *m*/*z* calcd for(C_27_H_25_N_3_OS + H)**^+^**: 440.1797; found: 440.1808.

*4-(2-(5-(4-bromophenyl)-3-(3,4-dichlorophenyl)-4,5-dihydro-1H-pyrazol-1-yl)thieno[3,2-d]pyrimidin-4-yl)morpholine* (**15b**). Light yellow solid, m.p. 265.3~266.3 °C; ^1^H NMR (400 MHz, DMSO-*d_6_*) δ (ppm): 7.69 (d, *J* = 7.8 Hz, 2H), 7.50 (d, *J* = 8.1 Hz, 2H), 7.40 (d, *J* = 6.2 Hz, 1H), 7.27 (d, *J* = 7.8 Hz, 2H), 7.20 (d, *J* = 7.8 Hz, 2H), 5.69 (dd, *J* = 12.2, 5.1 Hz, 1H), 3.93–3.82 (m, 1H), 3.62 (dd, *J* = 23.2, 8.0 Hz, 6H), 3.51 (d, *J* = 7.4 Hz, 2H), 3.12 (d, *J* = 18.1 Hz, 1H). HRMS (ESI): *m*/*z* calcd for (C_28_H_27_N_3_O_2_S + H)**^+^**: 470.1902; found: 470.1939.

*4-(2-(3,5-bis(4-fluorophenyl)-4,5-dihydro-1H-pyrazol-1-yl)thieno[3,2-d]pyrimidin-4-yl)Morpholine* (**15c**). Yellow solid, m.p. 254.7~255.4 °C; ^1^H NMR (400 MHz, DMSO-*d_6_*) δ (ppm): 7.88–7.82 (m, 2H), 7.39 (d, *J* = 6.1 Hz, 1H), 7.29 (dd, *J* = 16.3, 8.0 Hz, 4H), 7.19 (d, *J* = 6.1 Hz, 1H), 7.14 (d, *J* = 8.5 Hz, 2H), 5.73 (dd, *J* = 12.2, 5.2 Hz, 1H), 3.92–3.83 (m, 1H), 3.62 (dd, *J* = 20.8, 8.0 Hz, 6H), 3.51 (d, *J* = 8.0 Hz, 2H), 3.13 (dd, *J* = 18.0, 5.0 Hz, 1H). HRMS (ESI): *m*/*z* calcd for (C_27_H_23_F_2_N_3_OS + H)**^+^**: 476.1608; found: 476.1640.

*4-(2-(3-(3,4-dichlorophenyl)-5-(4-fluorophenyl)-4,5-dihydro-1H-pyrazol-1-yl)thieno[3,2-d]pyrmidin-4-yl)morpholine* (**15d**). Yellow solid, m.p. 237.7~238.8 °C; ^1^H NMR (400 MHz, DMSO-*d_6_*) δ (ppm): 7.60 (d, *J* = 7.9 Hz, 2H), 7.48 (d, *J* = 6.8 Hz, 2H), 7.31 (d, *J* = 5.8 Hz, 1H), 7.19 (d, *J* = 7.8 Hz, 2H), 7.11 (d, *J* = 6.4 Hz, 2H), 5.62 (d, *J* = 6.1 Hz, 1H), 3.82–3.73 (m, 1H), 3.55 (s, 2H), 3.51 (d, *J* = 7.4 Hz, 4H), 3.43 (d, *J* = 8.8 Hz, 2H), 3.08 (d, *J* = 12.8 Hz, 1H). ^13^C NMR (101 MHz, DMSO-*d_6_*) δ 162.70, 162.39, 161.13, 160.78, 157.90, 156.38, 140.04, 128.77, 128.03, 121.75, 115.91(2,C), 115.77(2,C), 114.63(3,C), 110.46, 66.28, 62.03, 55.83, 47.11, 42.77. HRMS (ESI): *m*/*z* calcd for (C_28_H_26_FN_3_O_2_S + H)**^+^**: 488.1808; found: 488.1842.

*4-(2-(3-(3,4-Dichlorophenyl)-5-phenyl-4,5-dihydro-1H-pyrazol-1-yl)thieno[3,2-d]pyrimidine-4-yl)morpholine* (**15e**). Yellow solid, m.p. 263–265 °C; ^1^H NMR (400 MHz, DMSO-*d_6_*) δ (ppm): 7.80 (d, *J* = 6.9 Hz, 2H), 7.61–7.55 (m, 2H), 7.49–7.44 (m, 3H), 7.43–7.39 (m, 1H), 7.21 (d, *J* = 6.2 Hz, 2H), 5.73 (dd, *J* = 12.2, 5.8 Hz, 1H), 3.89 (dd, *J* = 17.7, 12.2 Hz, 1H), 3.69–3.56 (m, 6H), 3.53–3.48 (m, 2H), 3.20 (dd, *J* = 17.8, 5.8 Hz, 1H). HRMS (ESI): *m*/*z* calcd for (C_28_H_26_BrN_3_OS + H)**^+^**: 534.1041; found: 534.1008.

*4-(2-(5-(4-methoxyphenyl)-3-phenyl-4,5-dihydro-1H-pyrazol-1-yl)thieno[3,2-d]pyrimidine-4-y)morpholine* (**15f**). Yellow solid, m.p. 235–237 °C; ^1^H NMR (400 MHz, DMSO-*d_6_*) δ (ppm): 7.78 (d, *J* = 8.5 Hz, 2H), 7.42 (d, *J* = 6.1 Hz, 1H), 7.30 (d, *J* = 7.2 Hz, 2H), 7.25 (d, *J* = 6.2 Hz, 4H), 7.04 (d, *J* = 8.6 Hz, 2H), 5.73–5.65 (m, 1H), 3.91 (dd, *J* = 18.0, 12.1 Hz, 1H), 3.82 (s, 3H), 3.66–3.53 (m, 6H), 3.46 (s, 2H), 3.16 (d, *J* = 17.8 Hz, 1H). HRMS (ESI): *m/z* calcd for (C_27_H_23_BrFN_3_OS + H)**^+^**: 538.0790; found: 538.0832.

*4-(2-(3-(3,4-dichlorophenyl)-5-(p-tolyl)-4,5-dihydro-1H-pyrazol-1-yl)thieno[3,2-d]pyrimidin-4-yl)morpholine* (**15g**). Yellow solid, m.p. 267–268 °C; ^1^H NMR (400 MHz, DMSO-*d_6_*) δ (ppm): 7.69 (d, *J* = 7.7 Hz, 2H), 7.57 (d, *J* = 8.2 Hz, 2H), 7.40 (d, *J* = 6.2 Hz, 1H), 7.28 (d, *J* = 8.0 Hz, 2H), 7.20 (d, *J* = 6.3 Hz, 2H), 5.70 (dd, *J* = 11.9, 5.6 Hz, 1H), 3.91–3.82 (m, 1H), 3.65–3.56 (m, 6H), 3.51 (d, *J* = 8.4 Hz, 2H), 3.21–3.13 (m, 1H), 2.35 (s, 3H). HRMS (ESI): *m*/*z* calcd for (C_27_H_24_BrN_3_OS + H)**^+^**: 517.0823; found: 517.0798.

*4-(2-(3-(4-bromophenyl)-5-(p-tolyl)-4,5-dihydro-1H-pyrazol-1-yl)thieno[3,2-d]pyrimidine-4-yl)morpholine* (**15h**). Yellow solid, m.p. 255–257 °C; ^1^H NMR (400 MHz, DMSO-*d_6_*) δ (ppm): 7.68 (d, *J* = 8.1 Hz, 2H), 7.50 (d, *J* = 8.3 Hz, 2H), 7.40 (t, *J* = 6.6 Hz, 1H), 7.29 (d, *J* = 10.6 Hz, 2H), 7.22 – 7.17 (m, 3H), 5.69 (dd, *J* = 11.7, 4.7 Hz, 1H), 3.92–3.83 (m, 1H), 3.63–3.55 (m, 6H), 3.50 (d, *J* = 11.1 Hz, 2H), 3.09 (dd, *J* = 17.6, 5.7 Hz, 1H), 2.35 (s, 3H). ^13^C NMR (101 MHz, DMSO-*d_6_*) δ 171.77, 158.31, 154.66, 151.51, 145.64, 132.28, 131.46, 131.41, 129.95, 129.76, 129.17(2,C), 128.46, 126.76(2,C), 126.26, 121.64, 117.97, 110.41, 66.32(2,C), 61.88, 46.96(2,C), 42.01.HRMS (ESI): *m*/*z* calcd for (C_27_H_23_Cl_2_N_3_OS + H)**^+^**: 508.1017; found: 508.1044.

*4-(2-(3,5-diphenyl-4,5-dihydro-1H-pyrazol-1-yl)thieno[3,2-d]pyrimidin-4-yl)morpholine* (**15i**). Yellow solid, m.p. 266–267 °C; ^1^H NMR (400 MHz, DMSO-*d_6_*) δ (ppm): 8.04 (d, *J* = 5.5 Hz, 1H), 7.77 (d, *J* = 7.6 Hz, 2H), 7.56–7.35 (m, 3H), 7.32–7.15 (m, 6H), 5.71 (dd, *J* = 12.0, 5.3 Hz, 1H), 3.87 (dd, *J* = 17.4, 12.2 Hz, 1H), 3.70–3.54 (m, 6H), 3.50 (s, 2H), 3.09 (dd, *J* = 17.6, 5.3 Hz, 1H). HRMS (ESI): *m*/*z* calcd for (C_27_H_22_Cl_2_FN_3_OS + H)**^+^**: 526.0923; found: 526.0889.

*4-(2-(3-(4-fluorophenyl)-5-(4-methoxyphenyl)-4,5-dihydro-1H-pyrazol-1-yl)thieno[3,2-d]pyrimidin-4-yl)morpholine* (**15j**). Yellow solid, m.p. 271–272 °C; ^1^H NMR (400 MHz, DMSO-*d_6_*) δ (ppm): 7.74 (d, *J* = 7.9 Hz, 2H), 7.35 (d, *J* = 6.0 Hz, 1H), 7.26–7.17 (m, 3H), 7.07 (d, *J* = 8.8 Hz, 2H), 6.95 (d, *J* = 8.6 Hz, 2H), 5.63 (dd, *J* = 11.8, 5.6 Hz, 1H), 3.85 (d, *J* = 17.8 Hz, 1H), 3.73 (s, 3H), 3.55 (dd, *J* = 25.4, 8.6 Hz, 7H), 3.09 (d, *J* = 16.8 Hz, 2H). HRMS (ESI): *m*/*z* calcd for (C_27_H_22_BrCl_2_N_3_OS + H)**^+^**: 588.0100; found: 588.0132.

*4-(2-(3-(4-bromophenyl)-5-(4-fluorophenyl)-4,5-dihydro-1H-pyrazol-1-yl)thieno[3,2-d]Pyrimidin-4-yl)morpholine* (**15k**). Yellow solid, m.p. 251–252 °C; ^1^H NMR (400 MHz, DMSO-*d_6_*) δ (ppm): 7.75 (s, 2H), 7.41 (d, *J* = 7.6 Hz, 2H), 7.31 (d, *J* = 5.5 Hz, 1H), 7.21 (t, *J* = 8.0 Hz, 2H), 7.12 (d, *J* = 7.9 Hz, 3H), 5.62 (d, *J* = 6.8 Hz, 1H), 3.85–3.74 (m, 1H), 3.53 (d, *J* = 13.2 Hz, 6H), 3.44 (d, *J* = 8.9 Hz, 2H), 3.05 (d, *J* = 17.7 Hz, 1H). HRMS (ESI): *m*/*z* calcd for (C_28_H_25_Cl_2_N_3_O_2_S + H)**^+^**: 538.1123; found: 538.1161.

*4-(2-(3-(4-bromophenyl)-5-phenyl-4,5-dihydro-1H-pyrazol-1-yl)thieno[3,2-d]pyrimidine-4-Morpholine* (**15l**). Yellow solid, m.p. 244–245 °C; ^1^H NMR (400 MHz, DMSO-*d_6_*) δ (ppm): 7.79 (d, *J* = 7.3 Hz, 2H), 7.50 (d, *J* = 8.3 Hz, 2H), 7.44 (t, *J* = 8.0 Hz, 3H), 7.40 (d, *J* = 6.2 Hz, 1H), 7.24–7.18 (m, 3H), 5.71 (dd, *J* = 12.1, 5.7 Hz, 1H), 3.89 (dd, *J* = 17.5, 12.6 Hz, 1H), 3.62 (dd, *J* = 23.5, 8.5 Hz, 6H), 3.51 (d, *J* = 8.0 Hz, 2H), 3.13 (dd, *J* = 17.7, 5.6 Hz, 1H). HRMS (ESI): *m*/*z* calcd for (C_28_H_25_Cl_2_N_3_OS + H)**^+^**: 522.1174; found: 522.1201.

4-(2-(3-(3,4-dichlorophenyl)-5-(4-methoxyphenyl)-4,5-dihydro-1H-pyrazol-1-yl)-5,6,7,8-tetrahydrobenzo[4,5]thieno[2,3-d]pyrimidin-4-yl)morpholine (**21a**). Dark blue solid, m.p. 252–253 °C; ESI-MS [M + H]^+^ *m*/*z*: 594.5; ^1^H NMR (400 MHz, DMSO-*d_6_*) δ (ppm): 7.73 (d, *J* = 8.7 Hz, 2H), 7.56 (d, *J* = 7.0 Hz, 2H), 7.20 (d, *J* = 8.4 Hz, 1H), 7.02 (d, *J* = 7.0 Hz, 2H), 5.67 (d, *J* = 5.7 Hz, 1H), 3.88 (d, *J* = 17.1 Hz, 1H), 3.81 (s, 3H), 3.62 (d, *J* = 5.4 Hz, 2H), 3.58 (s, 2H), 3.18 (d, *J* = 12.1 Hz, 3H), 2.99 (s, 2H), 2.73 (s, 4H), 1.81 (s, 4H).

4-(2-(3-(4-bromophenyl)-5-(p-tolyl)-4,5-dihydro-1H-pyrazol-1-yl)-5,6,7,8-tetrahdrobenzo[4,5]thieno[2,3-d]pyrimidin-4-yl)morpholine (**21b**). Dark blue solid, m.p. 243–244 °C; ESI-MS [M + H]^+^ *m*/*z*: 588.5; ^1^H NMR (400 MHz, DMSO-*d_6_*) δ (ppm): 7.69 (d, *J* = 7.9 Hz, 2H), 7.50 (d, *J* = 8.3 Hz, 2H), 7.27 (d, *J* = 7.7 Hz, 2H), 7.19 (d, *J* = 8.3 Hz, 2H), 5.70 (dd, *J* = 12.3, 5.4 Hz, 1H), 3.88 (dd, *J* = 17.7, 12.3 Hz, 1H), 3.64 (s, 2H), 3.57 (s, 2H), 3.15 (d, *J* = 16.2 Hz, 3H), 2.99 (s, 2H), 2.74 (s, 4H), 2.35 (s, 3H), 1.78 (d, *J* = 13.4 Hz, 4H).

4-(2-(3,5-diphenyl-4,5-dihydro-1H-pyrazol-1-yl)-5,6,7,8-tetrahydrobenzo[4,5]thiophene[2,3-d]pyramidin-4-yl)morpholine (**21c**). Dark blue solid, m.p. 212–213 °C; ESI-MS [M + H]^+^ *m*/*z*: 495.6; ^1^H NMR (400 MHz, DMSO-*d_6_*) δ (ppm): 7.82–7.79 (m, 2H), 7.46 (d, *J* = 7.6 Hz, 3H), 7.29 (d, *J* = 7.3 Hz, 2H), 7.23 (dd, *J* = 9.2, 1.9 Hz, 3H), 5.73 (dd, *J* = 12.1, 5.5 Hz, 1H), 3.92 (dd, *J* = 17.7, 12.1 Hz, 1H), 3.65–3.60 (m, 2H), 3.55 (d, *J* = 6.5 Hz, 2H), 3.14 (dd, *J* = 17.7, 5.7 Hz, 3H), 3.02–2.94 (m, 2H), 2.74 (s, 4H), 1.80 (d, *J* = 28.8 Hz, 4H).

4-(2-(3-(4-fluorophenyl)-5-(4-methoxyphenyl)-4,5-dihydro-1H-pyrazol-1-yl)-5,6,7,8-tetrahydrobenzo[4,5]thieno[2,3-d]pyrimidin-4-yl)morpholine (**21d**). Dark blue solid, m.p. 189-193 °C; ESI-MS [M + H]^+^ *m*/*z*: 543.6; ^1^H NMR (400 MHz, DMSO-*d_6_*) δ (ppm): 7.74 (d, *J* = 8.7 Hz, 2H), 7.28–7.23 (m, 2H), 7.12 (t, *J* = 8.8 Hz, 2H), 7.02 (d, *J* = 8.8 Hz, 2H), 5.71 (dd, *J* = 12.0, 5.4 Hz, 1H), 3.87 (d, *J* = 5.0 Hz, 1H), 3.81 (s, 3H), 3.68–3.61 (m, 2H), 3.57 (d, *J* = 6.4 Hz, 2H), 3.20–3.12 (m, 3H), 3.00 (s, 2H), 2.73 (s, 4H), 1.79 (d, *J* = 29.8 Hz, 4H).

4-(2-(3-(4-bromophenyl)-5-(4-fluorophenyl)-4,5-dihydro-1H-pyrazol-1-yl)-5,6,7,8-tetrahydrobenzo[4,5] thieno[2,3-d]pyrimidin-4-yl)morpholine (**21e**). Dark blue solid, m.p. 272–273 °C; ESI-MS [M + H]^+^ *m*/*z*: 592.5; ^1^H NMR (400 MHz, DMSO-*d_6_*) δ (ppm): 7.85 (d, *J* = 6.4 Hz, 2H), 7.50 (d, *J* = 7.3 Hz, 2H), 7.30 (s, 2H), 7.20 (d, *J* = 8.0 Hz, 2H), 5.77–5.66 (m, 1H), 3.91 (t, *J* = 16.3 Hz, 1H), 3.63 (s, 2H), 3.58 (s, 2H), 3.18 (s, 3H), 3.01 (s, 2H), 2.74 (s, 4H), 1.79 (d, *J* = 18.0 Hz, 4H).

4-(2-(3-(4-bromophenyl)-5-phenyl-4,5-dihydro-1H-pyrazol-1-yl)-5,6,7,8-tetrahydrobenzo[4,5]thieno[2,3-d]pyrimidin-4-yl)morpholine (**21f**). Dark blue solid, m.p. 257–259 °C; ESI-MS [M + H]^+^ *m*/*z*: 574.5; ^1^H NMR (400 MHz, DMSO-*d_6_*) δ (ppm): 7.80 (d, *J* = 7.8 Hz, 2H), 7.48 (dd, *J* = 17.6, 7.9 Hz, 5H), 7.20 (d, *J* = 8.4 Hz, 2H), 5.73 (dd, *J* = 11.9, 5.5 Hz, 1H), 3.91 (dd, *J* = 17.9, 12.1 Hz, 1H), 3.64 (d, *J* = 6.0 Hz, 2H), 3.57 (s, 2H), 3.15 (dd, *J* = 17.8, 5.5 Hz, 3H), 3.02 (s, 2H), 2.74 (s, 4H), 1.78 (d, *J* = 18.3 Hz, 4H).

4-(2-(5-(4-bromophenyl)-3-(3,4-dichlorophenyl)-4,5-dihydro-1H-pyrazol-1-yl)-5,6,7,8-tetrahydrobenzo[4,5]thieno[2,3-d]pyrimidin-4-yl)morpholine (**21g**). Dark blue solid, m.p. 231–234 °C; ESI-MS [M + H]^+^ *m*/*z*: 643.4; ^1^H NMR (400 MHz, DMSO-*d_6_*) δ (ppm): 7.71 (d, *J* = 8.3 Hz, 2H), 7.63 (d, *J* = 8.3 Hz, 2H), 7.57 (d, *J* = 11.3 Hz, 2H), 7.22 (d, *J* = 8.8 Hz, 1H), 5.71 (dd, *J* = 11.9, 5.9 Hz, 1H), 3.89 (dd, *J* = 18.0, 12.5 Hz, 1H), 3.62 (s, 2H), 3.57 (s, 2H), 3.21 (dd, *J* = 17.9, 6.2 Hz, 3H), 2.99 (s, 2H), 2.73 (s, 4H), 1.78 (d, *J* = 37.6 Hz, 4H).

4-(2-(3-(3,4-dichlorophenyl)-5-phenyl-4,5-dihydro-1H-pyrazol-1-yl)-5,6,7,8-tetrahydrobenzo[4,5]thieno[2,3-d]pyrimidin-4-yl)morpholine (**21h**). Dark blue solid, m.p. 293–294 °C; ESI-MS [M + H]^+^ *m*/*z*: 563.1; ^1^H NMR (400 MHz, DMSO-*d_6_*) δ (ppm): 7.80 (d, *J* = 6.5 Hz, 2H), 7.58 –7.55 (m, 2H), 7.46 (d, *J* = 7.6 Hz, 3H), 7.23–7.19(m, 1H), 5.73 (dd, *J* = 12.1, 5.9 Hz, 1H), 3.92 (dd, *J* = 17.8, 12.2 Hz, 1H), 3.63 (d, *J* = 6.2Hz, 2H), 3.58 (d, *J* = 6.4Hz, 2H), 3.21 (dd, *J* = 18.1, 6.3 Hz, 3H), 3.01 (s, 2H), 2.74 (s, 4H), 1.79 (d, *J* = 23.9 Hz, 4H).

### 3.3. Cytotoxicity Assay In Vitro

The *in vitro* cytotoxic activities of Compounds **9a**–**l**, **15a**–**l,** and **21a**–**h** were evaluated with A549, PC-3, HepG2, and MCF-7 cell lines by the standard MTT assay, with GDC-0941 as a positive control. The cancer cell lines were cultured in minimum essential medium (MEM) supplement with 10% fetal bovine serum (FBS). Approximately, 4 × 10 ^3^ cells, suspended in MEM medium, were plated onto each well of a 96-well plate and incubated in 5% CO_2_ at 37 °C for 24 h. The test compounds at the indicated final concentrations were added to the culture medium, and cell cultures continued for 72 h. Fresh MTT was added to each well at a terminal concentration of 5 µg/mL and incubated with cells at 37 °C for 4 h. The formazan crystals were dissolved in 100 µL of DMSO in each well, and the absorbency at 492 nm (for absorbance of MTT formazan) and 630 nm (for the reference wavelength) was measured with an enzyme linked immunosorbent assay (ELISA) reader (MR-96A Mindray Elisa Microplate Reader, Guangzhou, China). All compounds were tested three times in each of the cell lines. The results expressed as inhibition rates or IC_50_ was the averages of two determinations and calculated using the Bacus Laboratories Inc. Slide Scanner (Bliss) software (the Bacus Laboratories Inc. Slide Scanner (BLISS) system, Lombard, IL, USA).

### 3.4. mTOR Kinase Assay

The potent compounds **9a** and **15a** were tested for their activities against mTOR enzyme using Kinase-Glo^®^ Luminescent Kinase Assay (Promega, Madison, WI, USA), with NVPBEZ-235 and PI103 as positive controls. The kinase reaction was done in a 384-well black plate. Each well was loaded with 50 µL of test items (in 90% DMSO) and 5 µL reaction buffer containing 10 µg/mL PI substrate (l-α-phosphatidylinositol); Avanti Polar Lipids (Avanti Polar Lipids, Inc., Alabaster, AL, USA); prepared in 3% octyl-glucoside) and the mTOR protein 2.5 nM was then added to it. The reaction was started by the addition of 5 µL of 10 µM ATP prepared in the reaction buffer (50 mM HEPES pH 7.5, 1 mM EGTA, 3 mM MnCl_2_, 10 mM MgCl_2_, 2 mM DTT and 0.01% Tween-20) and was incubated for 60 min. It was terminated by the addition of 10 µL Kinase-Glo buffer. The plates were then read in Synergy 2 readers (BioTek, Winooski, VT, USA) for luminescence detection. The assay was repeated two times and the results expressed as IC_50_ (inhibitory concentration 50%) were the averages of two determinations.

### 3.5. PI3Kα Kinase Assay

The potent compounds **9a** and **15a** were tested for their activity against PI3Kα using a Kinase-Glo^®^ Luminescent Kinase Assay (Promega, Madison, WI, USA), with GDC-0941 and PI103 as positive controls. The kinase reaction occurred in a 384-well black plate. Each well was loaded with 50 µL of test items (in 90% DMSO) and 5 µL of reaction buffer containing 10µg/mL PI substrate (l-α-phosphatidylinositol; Avanti Polar Lipids (Avanti Polar Lipids, Inc.); prepared in 3% octyl-glucoside), and the PI3Kα protein (10 nM) was then added to it. The reaction was started by the addition of 5 µL of 1 µM Adenosine triphosphate (ATP) prepared in the reaction buffer and incubated for 60 min for p110α. It was terminated by the addition of 10 µL of Kinase-Glo buffer. The plates were then read in a Synergy 2 reader (BioTek, Winooski, VT, USA) for luminescence detection. The assay was repeated two times and the results expressed as IC_50_ (inhibitory concentration 50%) were the averages of two determinations.

### 3.6. Docking Studies

Molecular docking simulation studies were carried out by the AutoDock 4.2 software (The Scripps Research Institute, San Diego, CA, USA). The docking tutorial we used and the detailed AutoDock basic operational methods can be found at: http://autodock.scripps.edu/faqs-help/tutorial. The protein preparation process of flexible docking mainly includes fixing the exact residues, adding hydrogen atoms, removing irrelevant water molecules, adding charges, etc. The potent compounds were selected as ligand examples, and the structures of PI3Kα (PDB code: 3TL5, http://www.pdb.org/) were selected as the docking models. Only the best-scoring ligand–protein complexes were used for the binding site analyses. All the docking results were processed and modified in Open-Source PyMOL 1.8.x software (https://pymol.org).

## 4. Conclusions

Three series of thienopyrimidine derivatives containing pyrazole structure were designed and synthesized. In addition, we evaluated their cytotoxic activities against four cancer cell lines *in vitro*. The pharmacological indicated that most of the compounds showed moderate cytotoxic activity against the tested cancer cell lines. What is more, the inhibitory activities of the compounds **9a** and **15a** against PI3Kα and mTOR kinase were further investigated. Compound **9a** exhibited moderate levels of inhibition activity against four cancer cell lines and PI3Kα kinase. Although compound **9a** did not reach the same level of inhibition as the positive control drug, it also gave us new direction for developing novel thienopyrimidines containing the pyrazole linker group as PI3Kα inhibitor.

## Figures and Tables

**Table 1:**
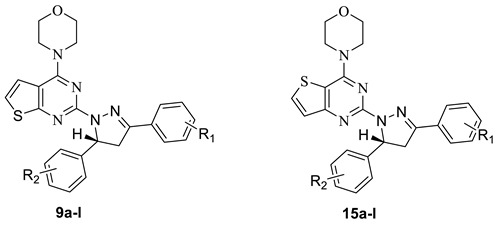
In vitro cytotoxic activities against different cancer cell lines of **9a**–**l** and **15a**–**l**.

Compd.	R_1_	R_2_		IC_50_ (μM) ^a^			ClogP ^b^	tPSA ^b^
			PC-3	A549	MCF-7	HepG2		
**9a**	4-H	4-H	12.32 ± 0.96	11.30 ± 1.19	14.69 ± 1.32	9.80 ± 0.93	6.67	28.07
**9b**	4-H	4-OCH_3_	15.36 ± 1.26	21.22 ± 2.75	14.12 ± 2.1	13.08 ± 1.20	6.59	37.3
**9c**	4-F	4-F	18.21 ± 1.55	24.23 ± 0.15	22.37 ± 0.17	17.36 ± 0.98	6.95	28.07
**9d**	4-F	4-OCH_3_	22.15 ± 1.67	>50	20.62 ± 1.90	17.98 ± 2.12	6.73	37.3
**9e**	4-Br	4-CH_3_	23.16 ± 1.96	>50	30.06 ± 2.86	21.63 ± 2.35	8.03	28.07
**9f**	4-Br	4-F	>50	15.37 ± 0.85	27.72 ± 2.71	13.49 ± 1.69	7.67	28.07
**9g**	4-Br	4-H	30.12 ± 3.01	22.78 ± 2.15	28.46 ± 3.9	28.85 ± 2.34	7.53	28.07
**9h**	3,4-di Cl	4-H	32.13 ± 2.96	38.53 ± 3.14	26.81 ± 2.13	23.70 ±2.08	7.97	28.07
**9i**	3,4-di Cl	4-F	27.65± 2.34	17.75 ± 1.64	29.53 ± 1.87	23.70 ± 2.11	8.12	28.07
**9j**	3,4-di Cl	4-Br	28.32 ± 2.16	>50	39.34 ± 2.51	>50	8.83	28.07
**9k**	3,4-di Cl	4-OCH_3_	33.54 ± 3.26	40.65 ± 3.85	25.30 ± 2.20	20.88 ± 1.98	7.89	37.3
**9l**	3,4-di Cl	4-CH_3_	28.36 ± 2.58	>50	>50	30.70 ± 0.15	8.47	28.07
**15a**	4-H	4-H	15.53 ± 1.21	16.90 ± 1.61	17.03 ± 1.68	13.14 ± 1.48	6.67	28.07
**15b**	4-H	4-OCH_3_	25.45 ± 2.32	>50	>50	34.62 ± 2.82	6.58	37.3
**15c**	4-F	4-F	25.31 ± 2.39	39.03 ± 3.32	37.12 ± 2.99	18.90 ± 1.86	6.95	28.07
**15d**	4-F	4-OCH_3_	22.13 ± 2.13	25.83 ± 1.99	35.15 ± 2.04	18.02 ± 1.30	6.73	37.3
**15e**	4-Br	4-CH_3_	37.12 ± 3.16	45.78 ± 0.81	>50	24.41 ± 1.14	8.03	28.07
**15f**	4-Br	4-F	26.45 ± 2.57	42.09 ± 0.08	36.17 ± 1.13	19.90 ± 1.10	7.67	28.07
**15g**	4-Br	4-H	24.22 ± 2.26	21.73 ± 1.47	32.14 ± 0.89	21.36 ± 1.86	7.53	28.07
**15h**	3,4-di Cl	4-H	27.15 ± 2.53	33.23 ± 2.14	>50	21.15 ± 1.97	7.97	28.07
**15i**	3,4-di Cl	4-F	18.48 ± 1.73	>50	>50	15.56 ± 1.30	8.12	28.07
**15j**	3,4-di Cl	4-Br	32.97 ±3.22	41.78 ± 0.81	>50	28.97 ± 2.49	8.83	28.07
**15k**	3,4-di Cl	4-OCH_3_	26.57 ± 2.38	31.05 ± 0.39	>50	19.86 ± 1.88	7.89	37.3
**15l**	3,4-di Cl	4-CH_3_	23.68 ±1.94	31.79 ± 0.52	>50	21.59 ± 1.86	8.47	28.07
**GDC-0941^c^**	-	-	4.35 ± 0.33	6.99 ± 0.21	0.20 ± 0.08	0.07 ± 0.03	3.20	76.85

The value “>50” indicates that no inhibitory effect at 50 μM compound concentration. ^a^ The values are an average of two separate determinations; ^b^ Calculated by ChemBioDraw Ultra 15.0; ^c^ used as a positive control.

**Table 2:**
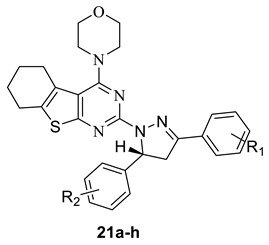
In vitro cytotoxic activities against different cancer cell lines of **21a**–**h**.

Compd.	R1	R2		IC_50_ (μM) ^a^			ClogP ^b^	tPSA ^b^
			PC-3	A549	MCF-7	HepG2		
**21a**	3,4-di Cl	4-OCH_3_	31.75 ± 0.95	27.68 ± 0.10	>501	32.37 ± 1.84	8.23	28.07
**21b**	4-Br	4-CH_3_	20.64 ± 0.63	11.59 ± 0.11	15.29 ± 0.83	12.43 ± 0.96	7.44	28.07
**21c**	4-H	4-H	>50	41.99 ± 1.49	>50	6>50	8.56	28.07
**21d**	4-F	4-OCH_3_	25.28 ± 0.75	17.75± 1.1	29.53 ± 1.87	23.70 ± 0.11	8.79	28.07
**21e**	4-Br	4-F	>50	>50	>50	38.71 ± 1.72	7.86	28.07
**21f**	4-Br	4-H	>50	>50	>50	>50	8.84	28.07
**21g**	3,4-diCl	4-Br	>50	>50	>50	>50	8.63	28.07
**21h**	3,4-diCl	4-H	>50	23.53 ± 0.82	>50	>50	7.95	28.07
**GDC-0941 ^c^**	-	-	4.35 ± 0.33	6.99 ± 0.21	0.20 ± 0.08	0.07 ± 0.03	3.20	76.85

The value “>50” indicates that no inhibitory effect at 50 μM compound concentration. ^a^ The values are an average of two separate determinations; ^b^ Calculated by ChemBioDraw Ultra 15.0; ^c^ used as a positive control.

**Table 3:** Enzymatic activities of compounds **9a** and **15a** against PI3Kα and mTOR (IC_50_, µM).

Compd.	R1	R2	IC_50_ (µM) ^a^	
			PI3Kα	mTOR
**9a**	4-H	4-H	9.47 ± 0.63	39.9 ± 7.6
**15a**	4-H	4-H	25.68 ± 2.33	>50
**PI-103 ^b^**	-	-	0.019 ± 0.004	0.011 ± 0.002

^a^ The values are an average of two separate determinations. ^b^ used as a positive control.
